# Does First‐Time Colon Cancer Screening Beyond Age 75 Reduce Cancer Risk Without Significant Complications? Insights From a Large US Retrospective Study

**DOI:** 10.1002/cam4.71200

**Published:** 2025-08-29

**Authors:** Azhar Hussain, Ruchir Paladiya, Muhammad Shahzil, Shiza Sarfraz, Kalsoom Khalil, Umar Hayat, Dushyant Singh Dahiya, Hassam Ali

**Affiliations:** ^1^ Department of Internal Medicine SUNY Upstate Medical University Syracuse New York USA; ^2^ Department of Internal Medicine University of Connecticut Health Center Farmington Connecticut USA; ^3^ Department of Internal Medicine Penn State Health Milton S Hershey Medical Center, The Pennsylvania State University Hershey Pennsylvania USA; ^4^ Department of Internal Medicine ECU Health Medical Center Greenville North Carolina USA; ^5^ Department of Internal Medicine Lahore General Hospital Lahore Pakistan; ^6^ Department of Internal Medicine Geisinger Wyoming Valley Medical Center Wilkes‐Barre Pennsylvania USA; ^7^ Division of Gastroenterology, Hepatology & Motility The University of Kansas School of Medicine Kansas City Kansas USA; ^8^ Department of Gastroenterology, Hepatology & Nutrition ECU Health Medical Center/Brody School of Medicine Greenville North Carolina USA

**Keywords:** aged 75 and over, colonoscopy, colorectal neoplasms, obesity, retrospective studies

## Abstract

**Introduction:**

Colorectal cancer (CRC) is a leading cause of global cancer‐related morbidity and mortality. This study examines the impact of first‐time screening colonoscopy on CRC incidence in obese individuals aged 75 and older using a large US retrospective cohort.

**Methods:**

Obese adults aged ≥ 75 were divided into two groups: those undergoing their first screening colonoscopy after 75 (Cases) and those without prior colonoscopy (Controls), using TrinetX database data.

**Results:**

Propensity‐matched cohorts of 123,930 patients each showed reduced CRC incidence (0.08% vs. 0.52%; OR: 0.157, *p* < 0.001) and lower all‐cause mortality (9.6% vs. 17.3%; OR: 0.510, *p* < 0.001) in the colonoscopy group. The number needed to treat to prevent one CRC case was 227. Perforation rates were similar (0.2%, *p* = NS), but gastrointestinal bleeding was higher (5.2% vs. 4.4%; OR: 1.187, *p* < 0.001).

**Conclusion:**

Screening colonoscopy after age 75 significantly reduces CRC incidence and mortality, with manageable complication rates.

## 1 Introduction

Colorectal cancer (CRC) continues to be a significant global health concern in individuals aged ≥ 75 years. In the United States, CRC is the third most common cancer and the second leading cause of cancer‐related mortality by 2024 [1]. The incidence rate of CRC in the > 75 years age group has been notably reported as 192.9 per 100,000 individuals [2]. The higher occurrence rate is affected by multiple factors, such as age‐related increases in the frequency of advanced colorectal polyps and comorbidities, which may contribute to CRC risk. A current screening rate of 68.8% has been reported for up‐to‐date CRC screening among adults aged 50–75 years [3]. The suboptimal screening rate among patients aged < 75 years increases the risk of CRC in patients aged > 75 years. As the geriatric population continues to rise, the medical and societal burden of CRC is expected to worsen over the years [4]. Current screening recommendations for CRC in individuals aged ≥ 75 years emphasize a personalized approach. The US Multi‐Society Task Force on CRC recommends that the decision to continue screening should be based on a shared decision‐making process that considers the patient's screening history, life expectancy, CRC risk, and personal preferences [5]. Screening is generally not recommended for individuals aged ≥ 86 years because of the increased risk of adverse events and limited potential benefits in terms of life expectancy. An observational study reported a lower risk of CRC incidence and CRC‐related mortality in individuals aged ≥ 75 years without significant comorbidities [6]. Obesity is also a significant public health issue in the United States, with a prevalence of 43% in adults [7]. Despite the well‐documented association between obesity and increased risk of CRC, there is limited evidence on the effectiveness of CRC screening in obese individuals aged 75 years and older. The USPSTF recommends that clinicians selectively offer screening for CRC in adults aged 76 to 85 years. Evidence indicates that the net benefit of screening all persons in this age group is small (Grade C Recommendation) [8].

We aimed to study this research gap by investigating the impact of first‐time screening colonoscopy on CRC incidence, mortality, and related outcomes in obese individuals aged > 75 years, using a large retrospective United States database, TriNetX.

## 2 Methods

This retrospective, propensity‐matched cohort study utilizes data from the TRINETX Research Network (Cambridge, MA, USA), which includes a cohort of 108 million patients from 69 healthcare organizations across the United States. All data used in this study are de‐identified and anonymized, with no patient, institutional, or geographical identifiers. The 69 contributing healthcare organizations include large academic medical centers and community hospitals. Data were collected from electronic health records (EHRs) and insurance claims, encompassing only inpatient settings to better reflect a real‐world clinical scenario and improve generalizability. A full description of the information about the data source, study definition, inclusion criteria, and exclusion criteria that were used to query the TRINETX Research Network database—and their associated ICD‐10 and CPT codes—are provided in the Supporting Information (appendix of study).

In this retrospective cohort, we analyzed obese adults aged 75 years and older, dividing them into two groups: those who underwent their first screening colonoscopy after the age of 75 with no prior reported colonoscopy (Cases) and those who did not (Controls). Exclusion criteria included a history of CRC, inflammatory bowel disease, Familial Adenomatous Polyposis, life expectancy < 1 year, incomplete data, and age ≤ 74 years. The primary objective was to determine the impact of first‐time screening colonoscopy on CRC incidence in obese individuals aged > 75 years. Secondary outcomes were all‐cause mortality and complications related to colonoscopy.

We used a propensity score matching algorithm to generate cohorts with matched demographics and baseline characteristics, aiming to decrease selection bias and control for confounding factors. The TrinetX built‐in propensity score matching function was applied at a 1:1 ratio by age at index event, gender, BMI, race, comorbidities, and lifestyle factors. A standardized difference of less than 0.1 was considered indicative of minimal differences between cohorts, suggesting effective matching. We then compared baseline demographic, laboratory, and clinical characteristics using either the Chi‐square/Fisher exact test or the independent sample *t*‐test. After propensity matching, odds ratios (ORs) with 95% confidence intervals (CIs) were calculated to compare outcomes between the cohorts, excluding patients who already had the outcome of interest prior to the index event. Kaplan–Meier curve analysis was also performed to demonstrate survival outcomes in both cohorts, and ORs with 95% CIs were calculated accordingly.

## 3 Results

After propensity score matching, each cohort included 123,930 patients. The mean age at index was similar between the groups (77.6 ± 2.8 years for the colonoscopy group vs. 77.7 ± 2.8 years for the no‐colonoscopy group, *p* < 0.001). The gender distribution was comparable (*p* < 0.001) (Supporting Information). Race distribution also showed slight differences in White race (*p* < 0.001), with no significant differences observed in other race categories such as Black or African American (12.3% vs. 12.4%, *p* = 0.534) or Native Hawaiian or Other Pacific Islander (1.3% in both groups, *p* = 0.740).

### 3.1 CRC Incidence and All‐Cause Mortality

**FIGURE 1:**
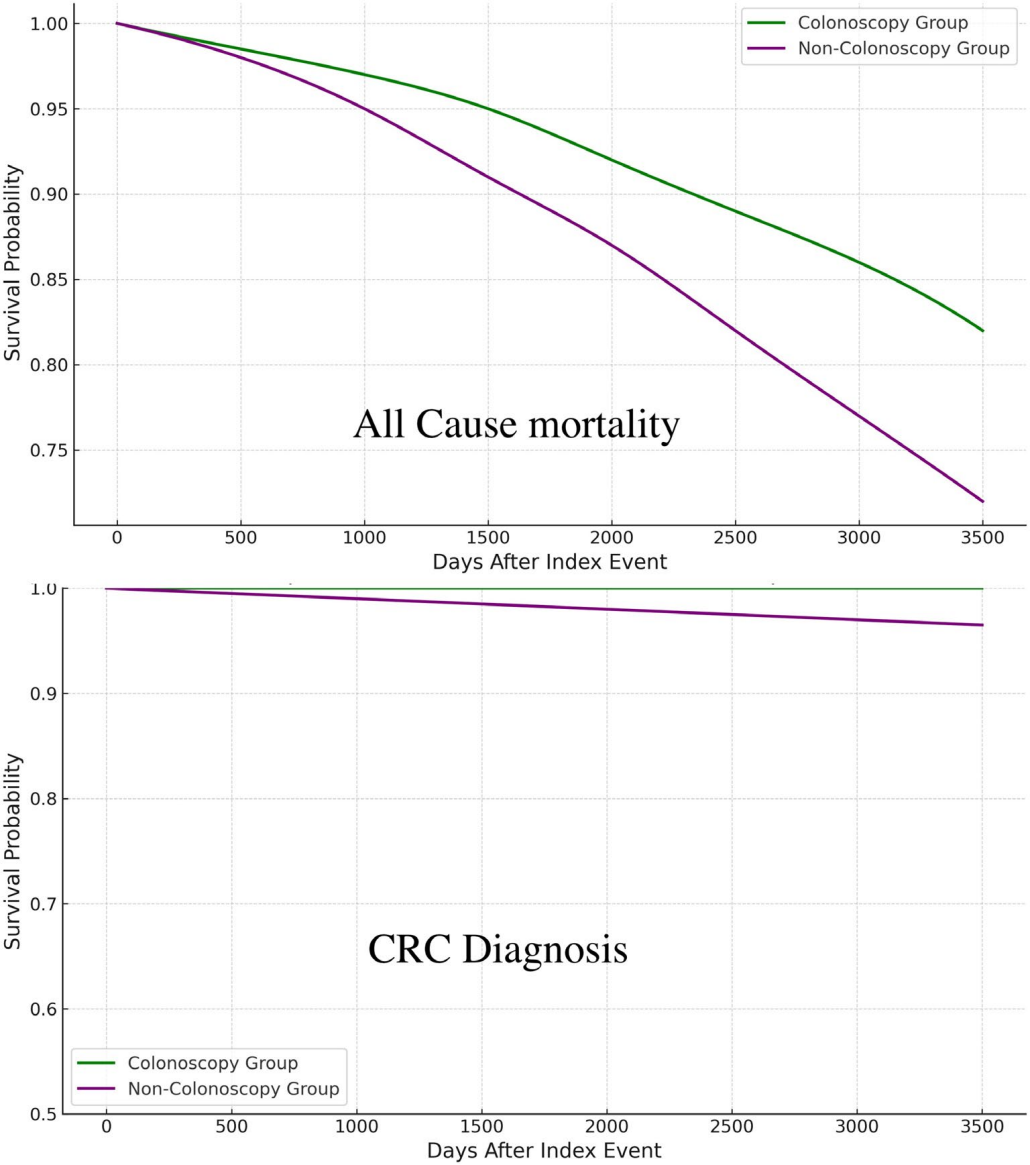
All‐cause mortality and colorectal cancer diagnosis in patients undergoing colon cancer screening (Purple) vs. no screening (Green) after the age of 75 years.

Colonoscopy significantly reduced the risk of CRC incidence (0.08% in the colonoscopy group vs. 0.52% in the no‐colonoscopy group; OR: 0.157, 95% CI: 0.127–0.194, *p* < 0.001). Similarly, all‐cause mortality was markedly lower in the colonoscopy group (9.6% vs. 17.3%; OR: 0.510, 95% CI: 0.498–0.522, *p* < 0.001). Kaplan–Meier survival analysis revealed a superior 5‐year survival probability in the colonoscopy group compared to the no‐colonoscopy group (61.75% vs. 47.60%, log‐rank *p* < 0.001) (Figure 1). The number needed to treat to prevent one case of colon cancer in this study population is approximately 227, indicating that for every 227 obese individuals aged 75 years or older who undergo first‐time colonoscopy screening, one case of colon cancer can be prevented.

### 3.2 Adverse Events and Complications

Gastrointestinal bleeding was higher in the colonoscopy group (5.2% vs. 4.4%; OR: 1.187, 95% CI: 1.144–1.231, *p* < 0.001), while rates of colon perforation were similar between the groups (0.2% in both cohorts; OR: 0.989, 95% CI: 0.832–1.176, *p* = 0.902). Atrial fibrillation occurred in 10.4% of patients in the colonoscopy group compared to 13.0% in the no‐colonoscopy group (OR: 0.777, 95% CI: 0.755–0.799, *p* < 0.001). The incidence of aspiration pneumonia was lower in the colonoscopy group (1.9% vs. 2.9%; OR: 0.663, 95% CI: 0.629–0.699, *p* < 0.001). Sepsis was less frequent in the colonoscopy group (6.1% vs. 8.3%; OR: 0.717, 95% CI: 0.695–0.741, *p* < 0.001), as was severe sepsis (1.9% vs. 2.6%; OR: 0.729, 95% CI: 0.690–0.769, *p* < 0.001). Acute kidney injury also occurred less frequently in the colonoscopy group (12.6% vs. 14.9%; OR: 0.820, 95% CI: 0.800–0.841, *p* < 0.001). Colon cancer resections were similar across groups (0.2% in both groups; OR: 0.936, 95% CI: 0.782–1.121, *p* = 0.474) (Table 1).

**TABLE 1:** Clinical outcomes between colonoscopy and no‐colonoscopy cohorts after propensity score matching.

Outcome	Colonoscopy	No colonoscopy	Risk ratio (95% CI)	Odds ratio (95% CI)	*p*
Colorectal cancer (CRC) incidence	100 (0.08%)	636 (0.52%)	NA	0.157 (0.127, 0.194)	0.000
All‐cause mortality	11,795 (9.6%)	21,161 (17.3%)	0.557 (0.546, 0.569)	0.510 (0.498, 0.522)	0.000
Colon‐Ca resection	230 (0.2%)	246 (0.2%)	0.937 (0.783, 1.121)	0.936 (0.782, 1.121)	0.474
Colon perforation	257 (0.2%)	260 (0.2%)	0.989 (0.833, 1.175)	0.989 (0.832, 1.176)	0.902
Gastrointestinal bleeding (GIB)	6443 (5.2%)	5474 (4.4%)	1.177 (1.136, 1.219)	1.187 (1.144, 1.231)	0.000
Atrial fibrillation (Afib)	10,001 (10.4%)	12,069 (13.0%)	0.800 (0.781, 0.820)	0.777 (0.755, 0.799)	0.000
Aspiration pneumonia	2380 (1.9%)	3556 (2.9%)	0.669 (0.636, 0.705)	0.663 (0.629, 0.699)	0.000
Sepsis	7094 (6.1%)	9593 (8.3%)	0.735 (0.713, 0.757)	0.717 (0.695, 0.741)	0.000
Severe sepsis	2336 (1.9%)	3175 (2.6%)	0.734 (0.696, 0.774)	0.729 (0.690, 0.769)	0.000
Acute kidney injury (AKI)	13,212 (12.6%)	15,625 (14.9%)	0.843 (0.825, 0.861)	0.820 (0.800, 0.841)	0.000

## 4 Discussion

The current study provides evidence that first‐time screening colonoscopy in obese individuals aged 75 years and older can significantly reduce the incidence of CRC and improve all‐cause mortality. These observations are important because older adults with obesity represent a population at heightened risk for CRC due to chronic inflammation, insulin resistance, and gut microbiota alterations that promote tumorigenesis [9]. Recent data have shown that obese individuals have an aOR of 1.47 for any colorectal neoplasm and 1.46 for advanced colorectal neoplasm [10]. Furthermore, obesity prevalence is disproportionately higher among African American and Hispanic older adults compared to non‐Hispanic whites, underscoring the need for targeted preventive strategies in these communities [11].

Despite guidelines suggesting individualization of screening in older adults, there is limited randomized or modeling evidence specifically supporting colonoscopy beyond 75 years, especially in the obese population. Our data address this gap by demonstrating not only a reduction in CRC incidence but also lower all‐cause mortality in individuals who underwent screening. The survival advantage at 5 years, illustrated by our Kaplan–Meier analysis, emphasizes the potentially transformative impact of continued screening in a traditionally under‐examined cohort. Early detection and removal of premalignant lesions likely contribute to reduced morbidity and mortality, aligning with the principle of targeted screening to avert progression to advanced disease.

The rates of complications such as atrial fibrillation, acute kidney injury, aspiration pneumonia, and sepsis‐related outcomes were all lower among those who underwent colonoscopy. While the procedure was associated with a modestly increased risk of gastrointestinal bleeding—a complication also noted in previous studies [12]—the overall benefit in survival appears to substantially outweigh this risk. Notably, colon cancer resection rates were similar in the colonoscopy and no‐colonoscopy groups, suggesting that screening leads to fewer advanced disease cases without increasing unnecessary surgical interventions. Recent research by Sepucha et al. emphasizes the importance of incorporating patient values and preferences in CRC screening decisions for older adults, aligning with a shared decision‐making approach [13]. While the relative reduction in CRC incidence is statistically significant, the absolute difference (0.08% vs. 0.52%) is small, and significance may be partly driven by the large cohort size. Nonetheless, the number needed to screen of 227 provided a clinically meaningful context for interpreting this benefit.

Our study has several limitations. First, it is a retrospective observational study, so no definitive causal inferences can be made. Second, inherent issues in EHRs—including potential overdiagnosis, underdiagnosis, and unmeasured confounders—may affect the results. Nevertheless, both Colonoscopy (+) and Colonoscopy (−) cohorts were drawn from the same standardized TriNetX database, which mitigates some biases by maintaining consistency in data collection. Third, while propensity score matching is adjusted for known confounders, unknown or unmeasured factors could still influence the outcomes. Full control of such confounding is only achievable through randomized controlled trials. Finally, although TriNetX encompasses approximately 28% of the US population, it may not fully represent the entire nation. Our findings should therefore be validated in broader and more diverse populations before being generalized. A key limitation is the lack of cancer‐specific mortality data in the TriNetX platform, preventing direct evaluation of CRC‐mortality. This limits our ability to definitively attribute the observed reduction in all‐cause mortality to CRC screening alone. Although we used robust propensity score matching to adjust for known confounders, residual confounding may persist. The all‐cause mortality benefit observed should be interpreted cautiously, especially given the lack of CRC‐specific mortality data and potential differences in unmeasured health behaviors, functional status, or frailty.

In conclusion, these results challenge the traditional age cutoff for CRC screening and highlight the need to reconsider current guidelines. Future prospective studies are warranted to refine risk‐stratification models and tailor screening intervals, ensuring that older, high‐risk individuals receive the greatest possible benefit from colon cancer screening.

## Author Contributions

**Azhar Hussain:** conceptualization, data curation, formal analysis, writing – original draft, writing – review and editing, software, funding acquisition. **Ruchir Paladiya:** data curation, writing – review and editing, writing – original draft, methodology. **Muhammad Shahzil:** writing – review and editing, writing – original draft, methodology, resources. **Shiza Sarfraz:** visualization, writing – review and editing, writing – original draft. **Kalsoom Khalil:** investigation, writing – review and editing, writing – original draft. **Umar Hayat:** data curation, supervision, writing – review and editing. **Dushyant Singh Dahiya:** writing – review and editing, writing – original draft, project administration, validation. **Hassam Ali:** supervision, conceptualization, writing – review and editing, project administration, validation.

## Ethics Statement

Institutional IRB approval was not obtained for this study as TrinetX is a third‐party de‐identified retrospective de‐identified database that is accessible for participating institutes.

## Consent

The authors have nothing to report.

## Conflicts of Interest

The authors declare no conflicts of interest.

## Supporting information


**Data S1:** cam471200‐sup‐0001‐TableS1‐FigureS1.docx.

## Data Availability

The datasets used and analyzed during the current study are not publicly available.
