# Supplementary material for: Does First‐Time Colon Cancer Screening Beyond Age 75 Reduce Cancer Risk Without Significant Complications? Insights From a Large US Retrospective Study
Source: Cancer Med. 2025 Aug 29;14(17):e71200. doi: 10.1002/cam4.71200 (PMC12395350; doi:10.1002/cam4.71200)
Supplement: Supplementary file 1 — Data S1: cam471200‐sup‐0001‐TableS1‐FigureS1.docx. [file CAM4-14-e71200-s001.docx]

Supplementary Table 1: Patient Characteristics and Laboratory valued before and after propensity score matching.

| **Variable & Category** | **Colonoscopy** | **No colonoscopy** | **P-Value** | **Colonoscopy** | **No Colonoscopy** | **P-Value** |
| --- | --- | --- | --- | --- | --- | --- |
| **Total** | 123,930 | 926,905 |  | 123,930 | 123,930 |  |
| **Age at Index event (Mean ± SD)** | 77.6 ± 2.8 | 75.0 ± 6.6, | <0.001 | 77.6 ± 2.8 | 77.7 ± 2.8 | <0.001 |
| **Gender** |  |  |  |  |  |  |
| **Female** | 64,420 (52.0%) | 508,325 (54.8%) | <0.001 | 64,420 (52.0%) | 65,384 (52.8%) | <0.001 |
| **Male** | 48,342 (39.0%) | 374,173 (40.4%) | <0.001 | 48,342 (39.0%) | 48,399 (39.1%) | 0.814 |
| **Race** |  |  |  |  |  |  |
| **White** | 89,495 (72.2%) | 683,476 (73.7%) | <0.001 | 89,495 (72.2%) | 90,322 (72.9%) | <0.001 |
| **Black or African American** | 15,302 (12.3%) | 94,594  (10.2%) | <0.001 | 15,302 (12.3%) | 15,404 (12.4%) | 0.534 |
| **Asian** | 1,657 (1.3%) | 10,570 (1.1%) | <0.001 | 1,657 (1.3%) | 1,673 (1.3%) | 0.780 |
| **American Indian or Alaska Native** | 246 (0.2%) | 2,259 (0.2%) | 0.002 | 246 (0.2%) | 259 (0.2%) | 0.563 |
| **Native Hawaiian or Other Pacific Islander** | 1,668 (1.3%) | 8,878 (1.0%) | <0.001 | 1,668 (1.3%) | 1,649 (1.3%) | 0.740 |
| **Other Race** | 2,447 (2.0%) | 22,479 (2.4%) | <0.001 | 2,447 (2.0%) | 2,455 (2.0%) | 0.908 |
| **Lab values (Mean ± SD)** |  |  |  |  |  |  |
| **Sodium [mmol/L]** | 139.6 ± 2.9 | 139.2 ± 3.2 | <0.001 | 139.6 ± 2.9 | 139.2 ± 3.2 | <0.001 |
| **Creatinine [mg/dL]** | 1.1 ± 1.7 | 1.1 ± 1.3 | <0.001 | 1.1 ± 1.7 | 1.1 ± 1.1 | <0.001 |
| **Platelets [10^3/uL]** | 231.6 ± 71.7 | 230.4 ± 77.9 | <0.001 | 231.6 ± 71.7 | 229.4 ± 77.6 | <0.001 |
| **Alanine aminotransferase [U/L]** | 21.6 ± 22.1 | 24.8 ± 42.6 | <0.001 | 21.6 ± 22.1 | 23.6 ± 34.5 | <0.001 |
| **Aspartate aminotransferase [U/L]** | 23.4 ± 19.1 | 26.3 ± 76.4 | <0.001 | 23.4 ± 19.1 | 26.1 ± 120.7 | <0.001 |
| **Bilirubin.total [mg/dL]** | 0.6 ± 0.4 | 0.6 ± 0.7 | <0.001 | 0.6 ± 0.4 | 0.6 ± 0.6 | <0.001 |
| **Albumin [g/dL]** | 4.0 ± 0.5 | 3.8 ± 0.6 | <0.001 | 4.0 ± 0.5 | 3.8 ± 0.5 | <0.001 |
| **Protein [g/dL]** | 6.9 ± 0.7 | 6.9 ± 0.8 | <0.001 | 6.9 ± 0.7 | 6.9 ± 0.7 | <0.001 |
| **Prothrombin time (PT) [s]** | 14.2 ± 6.0 | 14.3 ± 6.0 | <0.001 | 14.2 ± 6.0 | 14.6 ± 5.8 | <0.001 |
| **Activated partial thromboplastin time (aPTT) [s]** | 32.4 ± 12.4 | 32.2 ± 12.3 | <0.001 | 32.4 ± 12.4 | 32.8 ± 12.8 | <0.001 |
| **INR** | 1.2 ± 0.6 | 1.3 ± 0.7 | <0.001 | 1.2 ± 0.6 | 1.3 ± 0.7 | <0.001 |
| **BMI [kg/m^2]** | 32.8 ± 5.6 | 33.8 ± 6.2 | <0.001 | 32.8 ± 5.6 | 33.5 ± 5.9 | 0.877 |

Supplementary Figure 1: Comparison of outcomes between colonoscopy and no colonoscopy groups in patients above age 75 years with obesity

**
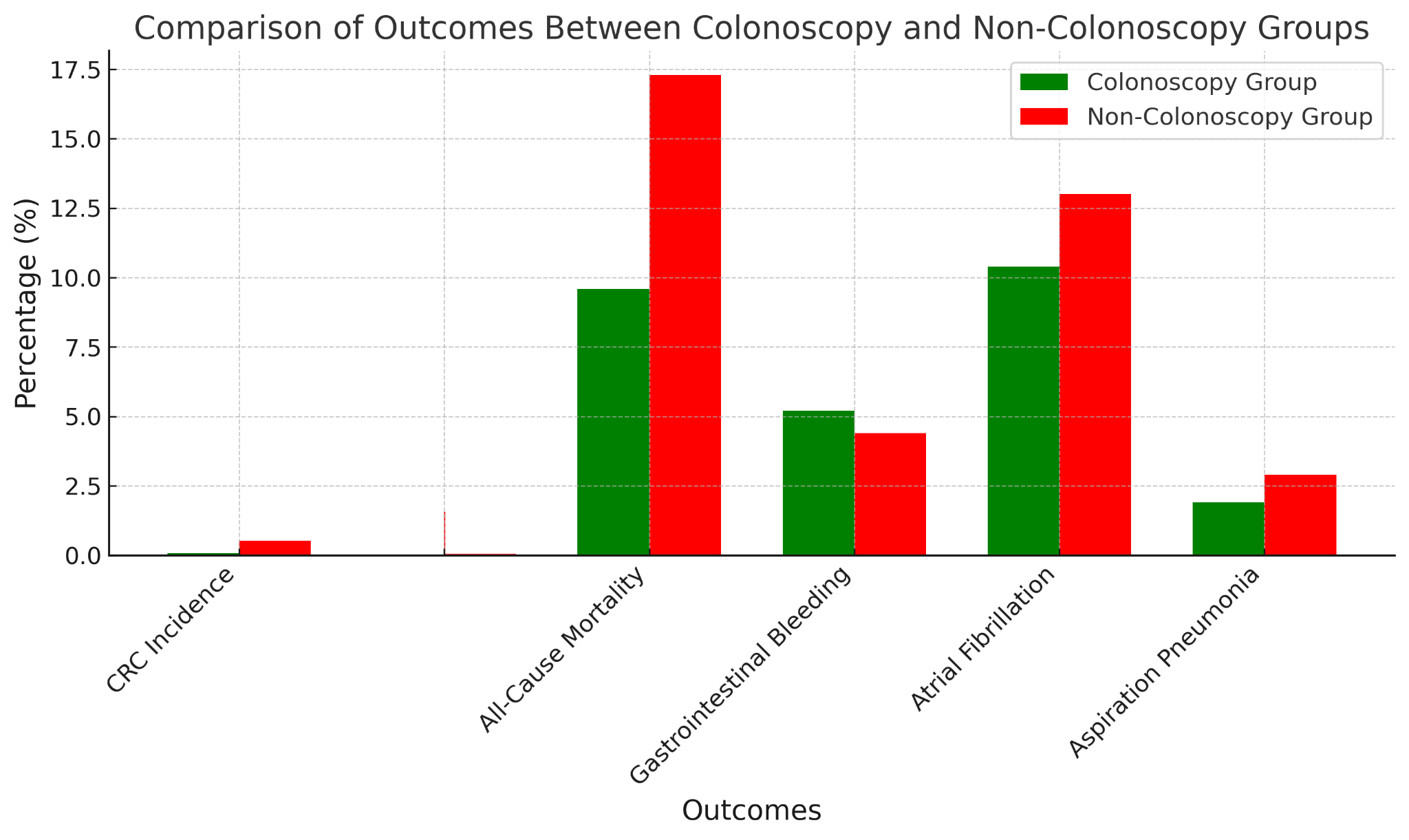
**

# Appendix A – Text Representation of the Cohorts Definition

This section lists all terms used in the definitions of the two cohorts.

### Query Criteria for Cohort 1 (query name: Obese+Colonoscopy)

Patients must have:
 all of the following:
 Age (Age) (at least 75 years (most recent occurrence)); and
 any of the following:
 Colonoscopy, flexible; with removal of tumor(s), polyp(s), or other lesion(s) by hot biopsy forceps (UMLS:CPT:45384) (at least 75 years old at event); or
 Colonoscopy, flexible; with removal of tumor(s), polyp(s), or other lesion(s) by snare technique (UMLS:CPT:45385) (at least 75 years old at event); or
 Colonoscopy, flexible; with ablation of tumor(s), polyp(s), or other lesion(s) (includes pre- and post-dilation and guide wire passage, when performed) (UMLS:CPT:45388) (at least 75 years old at event); or
 Colorectal cancer screening; colonoscopy on individual at high risk (UMLS:HCPCS:G0105) (at least 75 years old at event); or
 Colorectal cancer screening; colonoscopy on individual not meeting criteria for high risk (UMLS:HCPCS:G0121) (at least 75 years old at event); or
 Encounter for screening for malignant neoplasm of colon (UMLS:ICD10CM:Z12.11) (at least 75 years old at event); and
 Overweight and obesity (UMLS:ICD10CM:E66).

Patients cannot have:
 any of the following:
 Malignant neoplasm of colon (UMLS:ICD10CM:C18); or
 Malignant neoplasm of rectosigmoid junction (UMLS:ICD10CM:C19); or
 Malignant neoplasm of rectum (UMLS:ICD10CM:C20); or
 Crohn's disease [regional enteritis] (UMLS:ICD10CM:K50); or
 Ulcerative colitis (UMLS:ICD10CM:K51); or
 Familial adenomatous polyposis (UMLS:ICD10CM:D13.91); or
 Genetic susceptibility to other malignant neoplasm (UMLS:ICD10CM:Z15.09); or
 Genetic carrier of other disease (UMLS:ICD10CM:Z14.8).

### Query Criteria for Cohort 2 (query name: Obese-Colonoscopy)

Patients must have:
 all of the following:
 Age (Age) (at least 75 years (most recent occurrence)); and
 Overweight and obesity (UMLS:ICD10CM:E66).

Patients cannot have:
 any of the following:
 Malignant neoplasm of colon (UMLS:ICD10CM:C18) (at most 74 years old at event); or
 Colorectal cancer screening; colonoscopy on individual at high risk (UMLS:HCPCS:G0105); or
 Colorectal cancer screening; colonoscopy on individual not meeting criteria for high risk (UMLS:HCPCS:G0121); or
 Colonoscopy, flexible; with ablation of tumor(s), polyp(s), or other lesion(s) (includes pre- and post-dilation and guide wire passage, when performed) (UMLS:CPT:45388); or
 Colonoscopy, flexible; with removal of tumor(s), polyp(s), or other lesion(s) by snare technique (UMLS:CPT:45385); or
 Colonoscopy, flexible; with removal of tumor(s), polyp(s), or other lesion(s) by hot biopsy forceps (UMLS:CPT:45384); or
 Malignant neoplasm of rectosigmoid junction (UMLS:ICD10CM:C19) (at most 74 years old at event); or
 Malignant neoplasm of rectum (UMLS:ICD10CM:C20) (at most 74 years old at event); or
 Crohn's disease [regional enteritis] (UMLS:ICD10CM:K50); or
 Ulcerative colitis (UMLS:ICD10CM:K51); or
 Familial adenomatous polyposis (UMLS:ICD10CM:D13.91); or
 Genetic susceptibility to other malignant neoplasm (UMLS:ICD10CM:Z15.09); or
 Genetic carrier of other disease (UMLS:ICD10CM:Z14.8); or
 Encounter for screening for malignant neoplasm of colon (UMLS:ICD10CM:Z12.11).

# Appendix B – Text Representation of the Analysis Setup

This section contains the Index Event definition for each cohort.

The index event for Cohort 1 (query name: Obese+Colonoscopy) is defined as the following:

Patients must have:
 all of the following:
 any of the following:
 Colonoscopy, flexible; with removal of tumor(s), polyp(s), or other lesion(s) by hot biopsy forceps (UMLS:CPT:45384) (at least 75 years old at event); or
 Colonoscopy, flexible; with removal of tumor(s), polyp(s), or other lesion(s) by snare technique (UMLS:CPT:45385) (at least 75 years old at event); or
 Colonoscopy, flexible; with ablation of tumor(s), polyp(s), or other lesion(s) (includes pre- and post-dilation and guide wire passage, when performed) (UMLS:CPT:45388) (at least 75 years old at event); or
 Colorectal cancer screening; colonoscopy on individual at high risk (UMLS:HCPCS:G0105) (at least 75 years old at event); or
 Colorectal cancer screening; colonoscopy on individual not meeting criteria for high risk (UMLS:HCPCS:G0121) (at least 75 years old at event); or
 Encounter for screening for malignant neoplasm of colon (UMLS:ICD10CM:Z12.11) (at least 75 years old at event); and
 Overweight and obesity (UMLS:ICD10CM:E66).

The index event for Cohort 2 (query name: Obese-Colonoscopy) is defined as the following:

Patients must have:
 Overweight and obesity (UMLS:ICD10CM:E66).

# Appendix C – Text Representation of the Outcomes Definition

This analysis includes the following outcomes:

Sepsis
 Patients must have:
 Other sepsis (UMLS:ICD10CM:A41).

Severe sepsis
 Patients must have:
 Severe sepsis without septic shock (UMLS:ICD10CM:R65.20).

HE
 Patients must have:
 any of the following:
 Hepatic encephalopathy (UMLS:ICD10CM:K76.82); or
 Hepatic failure, unspecified with coma (UMLS:ICD10CM:K72.91).

AKI
 Patients must have:
 any of the following:
 Acute kidney failure (UMLS:ICD10CM:N17); or
 Acute kidney failure with tubular necrosis (UMLS:ICD10CM:N17.0); or
 Acute kidney failure, unspecified (UMLS:ICD10CM:N17.9); or
 Other acute kidney failure (UMLS:ICD10CM:N17.8); or
 Acute kidney failure with acute cortical necrosis (UMLS:ICD10CM:N17.1); or
 Acute kidney failure with medullary necrosis (UMLS:ICD10CM:N17.2).


CRC
 Patients must have:
 any of the following:
 Malignant neoplasm of colon (UMLS:ICD10CM:C18); or
 Malignant neoplasm of rectosigmoid junction (UMLS:ICD10CM:C19); or
 Malignant neoplasm of rectum (UMLS:ICD10CM:C20).

all-cause mortality
 Patients must have:
 any of the following:
 Deceased (Deceased); or
 Ill-defined and unknown cause of mortality (UMLS:ICD10CM:R99).

Colon-Ca Resection
 Patients must have:
 any of the following:
 Resection of Ascending Colon, Percutaneous Endoscopic Approach (UMLS:ICD10PCS:0DTK4ZZ); or
 Resection of Descending Colon, Percutaneous Endoscopic Approach (UMLS:ICD10PCS:0DTM4ZZ); or
 Resection of Descending Colon, Via Natural or Artificial Opening Endoscopic (UMLS:ICD10PCS:0DTM8ZZ); or
 Partial resection of colon (UMLS:SNOMED:43075005); or
 Excision of Sigmoid Colon, Open Approach (UMLS:ICD10PCS:0DBN0ZZ); or
 Gastrointestinal System / Resection / Ascending Colon (UMLS:ICD10PCS:0DTK); or
 Excision of Large Intestine, Open Approach (UMLS:ICD10PCS:0DBE0ZZ); or
 Gastrointestinal System / Resection / Transverse Colon (UMLS:ICD10PCS:0DTL); or
 Gastrointestinal System / Resection / Descending Colon (UMLS:ICD10PCS:0DTM); or
 Resection of Sigmoid Colon, Open Approach (UMLS:ICD10PCS:0DTN0ZZ); or
 Resection of Ascending Colon, Open Approach (UMLS:ICD10PCS:0DTK0ZZ); or
 Resection of Transverse Colon, Open Approach (UMLS:ICD10PCS:0DTL0ZZ); or
 Resection of Descending Colon, Open Approach (UMLS:ICD10PCS:0DTM0ZZ); or
 Resection of Transverse Colon, Percutaneous Endoscopic Approach (UMLS:ICD10PCS:0DTL4ZZ); or
 Partial resection of colon with stoma (UMLS:SNOMED:738552004); or
 Resection of Sigmoid Colon, Percutaneous Endoscopic Approach (UMLS:ICD10PCS:0DTN4ZZ); or
 Resection of Ascending Colon, Percutaneous Endoscopic Approach (UMLS:ICD10PCS:0DTK4ZZ); or
 Resection of Descending Colon, Percutaneous Endoscopic Approach (UMLS:ICD10PCS:0DTM4ZZ).

Colon perforation
 Patients must have:
 any of the following:
 Perforation of intestine (nontraumatic) (UMLS:ICD10CM:K63.1); or
 Unspecified injury of sigmoid colon, initial encounter (UMLS:ICD10CM:S36.503A); or
 Unspecified injury of transverse colon, initial encounter (UMLS:ICD10CM:S36.501A); or
 Unspecified injury of ascending [right] colon, initial encounter (UMLS:ICD10CM:S36.500A); or
 Unspecified injury of descending [left] colon, initial encounter (UMLS:ICD10CM:S36.502A).

GIB
 Patients must have:
 Gastrointestinal hemorrhage, unspecified (UMLS:ICD10CM:K92.2).

A.fib
 Patients must have:
 Atrial fibrillation and flutter (UMLS:ICD10CM:I48).

PNA, aspiration
 Patients must have:
 Pneumonitis due to inhalation of food and vomit (UMLS:ICD10CM:J69.0).
